# PlantPhos: using maximal dependence decomposition to identify plant phosphorylation sites with substrate site specificity

**DOI:** 10.1186/1471-2105-12-261

**Published:** 2011-06-26

**Authors:** Tzong-Yi Lee, Neil Arvin Bretaña, Cheng-Tsung Lu

**Affiliations:** 1 Department of Computer Science and Engineering, Yuan Ze University, Chungli 320, Taiwan

## Abstract

**Background:**

Protein phosphorylation catalyzed by kinases plays crucial regulatory roles in intracellular signal transduction. Due to the difficulty in performing high-throughput mass spectrometry-based experiment, there is a desire to predict phosphorylation sites using computational methods. However, previous studies regarding *in silico *prediction of plant phosphorylation sites lack the consideration of kinase-specific phosphorylation data. Thus, we are motivated to propose a new method that investigates different substrate specificities in plant phosphorylation sites.

**Results:**

Experimentally verified phosphorylation data were extracted from TAIR9-a protein database containing 3006 phosphorylation data from the plant species *Arabidopsis thaliana*. In an attempt to investigate the various substrate motifs in plant phosphorylation, maximal dependence decomposition (MDD) is employed to cluster a large set of phosphorylation data into subgroups containing significantly conserved motifs. Profile hidden Markov model (HMM) is then applied to learn a predictive model for each subgroup. Cross-validation evaluation on the MDD-clustered HMMs yields an average accuracy of 82.4% for serine, 78.6% for threonine, and 89.0% for tyrosine models. Moreover, independent test results using *Arabidopsis thaliana *phosphorylation data from UniProtKB/Swiss-Prot show that the proposed models are able to correctly predict 81.4% phosphoserine, 77.1% phosphothreonine, and 83.7% phosphotyrosine sites. Interestingly, several MDD-clustered subgroups are observed to have similar amino acid conservation with the substrate motifs of well-known kinases from Phospho.ELM-a database containing kinase-specific phosphorylation data from multiple organisms.

**Conclusions:**

This work presents a novel method for identifying plant phosphorylation sites with various substrate motifs. Based on cross-validation and independent testing, results show that the MDD-clustered models outperform models trained without using MDD. The proposed method has been implemented as a web-based plant phosphorylation prediction tool, PlantPhos http://csb.cse.yzu.edu.tw/PlantPhos/. Additionally, two case studies have been demonstrated to further evaluate the effectiveness of PlantPhos.

## Background

Protein phosphorylation is the most widespread and well-studied post-translational modification in eukaryotic cells. It is one of the most prevalent intracellular protein modifications that influence numerous cellular processes [1]. It has been estimated that one-third to one-half of all proteins in a eukaryotic cell are phosphorylated [2]. Furthermore, protein phosphorylation, catalyzed by specific kinases, plays crucial regulatory roles in intracellular signal transduction. Networks composed of proteins and small molecules that transmit information from the cell surface to the nucleus are ultimately affected by transcriptional changes [3]. An estimated 1% to 3% of functional eukaryotic genes encode protein kinases; this suggests that they are involved in many aspects of cellular regulation and metabolism [4]. However, a full understanding on the mechanism of intracellular signal transduction remains a major challenge in cellular biology.

Protein phosphorylation regulates various cellular processes not only in mammals but also in plants. It is reported that the regulation of carbon and nitrogen metabolism in plants is driven by phosphorylation [5]. Phosphorylation is also involved in modulating a sucrose phosphate synthase enzyme which controls the signaling pathway for the process of sucrose synthesis in plants [6]. Phosphorylation also aids in modulating the plant process of synthesizing Ammonia, an organic compound which is required to give energy to certain organs which are not able to photosynthesize [6]. Furthermore, protein phosphorylation is involved in the process of plant growth and plant response to stress [6,7]. Stone *et al. *have identified a number of plant kinases; however, the precise functional roles of specific protein kinases were not widely elucidated [4].

Due to the interest of the scientific community in further understanding the process of phosphorylation, mass spectrometry-based proteomics have been used to enable the large-scale mapping of *in vivo *phosphorylation sites [8]. With this, several databases have been proposed to store experimentally verified phosphorylation sites with catalytic kinases, such as Phospho.ELM [9], PhosphoSite [10], UniProtKB/Swiss-Prot [11], PHOSIDA [12], and dbPTM [13,14]. While most resources focus on phosphorylation sites in mammalian organisms, there are some databases which store phosphorylation sites in plants such as PhosPhAt [15], P3DB [16] and TAIR [17]. PhosPhAt consolidates knowledge of mass spectrometry-based identified phosphorylation sites in *Arabidopsis thaliana *and offers a phosphorylation site prediction tool specifically trained on experimentally identified *Arabidopsis thaliana *phosphorylation motifs [15]. P3DB provides a resource of protein phosphorylation data from multiple plants. Moreover, a phosphopeptide BLAST browser was implemented to allow users to query the database for phosphopeptides similar to protein sequences of their interest [16]. TAIR maintains a database of genetic and molecular data for *Arabidopsis thaliana *[17]. Protein data stored in TAIR includes the complete protein sequence along with phosphorylation site annotations.

Due to the high complexity and difficulty of phosphorylation site identification using mass spectrometry, a number of mammalian protein phosphorylation prediction tools have been developed using different methods and yielding various predictive performance. KinasePhos 1.0 [18,19], incorporated profile HMM for identifying kinase-specific phosphorylation site prediction, whose overall predictive accuracy is about 87%. Version 2.0 of KinasePhos [20] incorporated support vector machine (SVM) with the protein coupling pattern to identify phosphorylation sites for 58 kinase groups. NetPhosK [21] applied an artificial neural network algorithm to predict 17 PK groups-specific phosphorylation sites. Scansite 2.0 [22] identified short protein sequence motifs that are recognized by modular signaling domains, phosphorylated by serine/threonine, tyrosine kinases or those that mediate specific interactions with protein or phospholipid ligands. GPS [23,24] is a group-based phosphorylation site prediction and scoring platform which clusters 216 unique protein kinases in 71 groups. PPSP [25] developed an approach based on Bayesian decision theory for predicting the potential phosphorylation sites accurately for around 70 protein kinase groups. PHOSIDA [12], incorporated SVM with surface accessibility and evolutionary conservation, made 91.75%, 81.06%, and 76.19% accuracies in serine, threonine, and tyrosine, respectively.

With regard to plant phosphorylation prediction, PhosPhAt [15] has utilized a set of 802 experimentally validated phosphoserine sites to develop a classifier of SVM for identifying pSer sites in *Arabidopsis thaliana*. This yielded an area under curve rate of around 0.81 on a redundant TAIR7 [17] protein dataset. More recently, Gao *et al. *[26] incorporated protein sequence information and protein disordered regions, and integrated k-nearest neighbor and SVM for predicting phosphorylation sites. Their method utilized the PhosPhAt dataset of pSer in *Arabidopsis thaliana *and the TAIR7 non-redundant protein database. However, these works do not predict phosphorylation sites according to plant substrate site specificity [26]. Therefore, there is a need to investigate the various substrate site specificities in plants and utilize this information for predicting kinase-specific plant protein phosphorylation sites.

Information regarding protein kinases that phosphorylate substrates in plants is very limited. Based on the collection of experimentally verified plant phosphorylation sites from TAIR9 and UniProtKB/Swiss-Prot, phosphorylation sites are not annotated with its corresponding kinase. Due to this limitation, majority of the published methods for computationally identifying kinase-specific phosphorylation sites are trained mainly by using data from non-plant organisms. This study aims to analyze plant phosphorylation sites, investigate substrate site specificity in plants, and most importantly, present a novel method for identifying potential phosphorylation sites in plant proteins using the available substrate site specificity information. This work applies maximal dependence decomposition (MDD) [27] to cluster all phosphorylated fragments into subgroups presenting meaningful and statistically significant site specificity. MDD was firstly used to group the splice sites during the identification process of splice site prediction [28]. A large group of aligned sequences can be moderated into subgroups that capture the most significant dependencies between positions. Huang *et al. *[19] have applied MDD to improve the prediction performance of PKA, PKC, and CK2 kinase groups. In this study, MDD is adopted to investigate various substrate specificities of plant phosphorylation sites. Additionally, the motif of each MDD-clustered subgroup is compared to the substrate motifs of known kinases in Phospho.ELM [9]-a database for integrating comprehensive information of kinase-specific phosphorylation sites from multiple organisms. According to a five-fold cross-validation evaluation, models trained with MDD-clustered subgroups could improve predictive accuracy as compared to models trained without the application of MDD clustering. Furthermore, an independent data set is used to further evaluate the effectiveness of the models that achieve the best accuracy during cross-validation. Finally, the MDD-clustered models are adopted to implement an effective web-based tool, namely PlantPhos, for identifying plant phosphorylation sites with substrate motifs that may potentially be recognized by plant kinases. The prediction tool and the data used in this study can be available at http://csb.cse.yzu.edu.tw/PlantPhos/.

## Materials and methods

**Figure 1:**
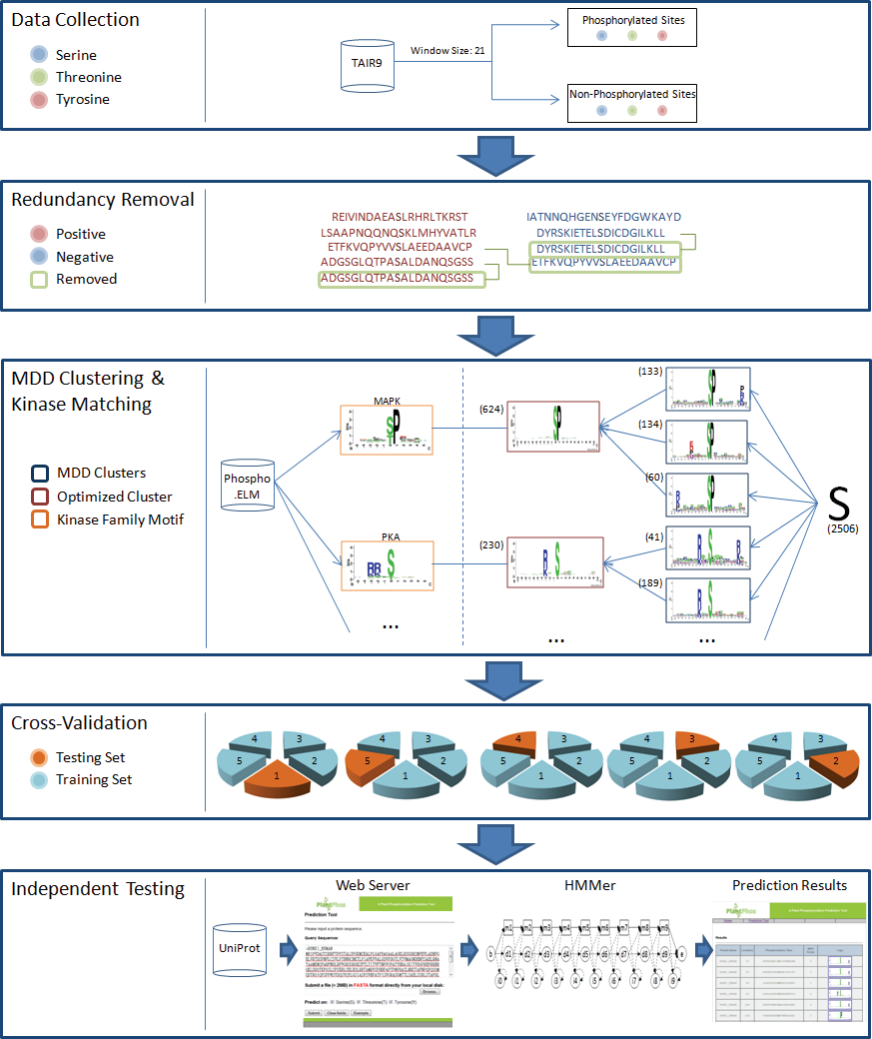
**System flow of this study**.

Figure 1 depicts the system flow of this study. The method consists of the following processes: data collection, redundancy removal, data clustering by MDD, model learning and cross-validation, and independent testing. The details of each process are described as follows.

### Data collection and preprocessing

With reference to PhosPhAt [15], this work obtains plant protein phosphorylation data from the TAIR9 database [17]. TAIR9 consists of 3006 phosphorylation data from the plant species *Arabidopsis thaliana*. The database contains annotations of the phosphorylated sites in each plant protein data. Based on the concept of binary classification, the residues serine (S), threonine (T), and tyrosine (Y) which are annotated as phosphorylation sites in the database are regarded as positive data. On the other hand, with reference to previous works [18,26], S, T, and Y residues which are not annotated as phosphorylated are extracted as negative data. In order to investigate the surrounding residues of phosphorylation sites in a comprehensive manner, sequence fragments are extracted using a window size of 21 with S, T, or Y as the central residue. As presented in Table 1, this resulted in 2516 positive data and 97965 negative data for S; 382 positive data and 51434 negative data for T; and 108 positive data and 26405 negative data for Y.

**Table 1:** Data statistics of the experimentally verified phosphorylation sites collected from TAIR9 protein database.

Phosphorylated residue	Type of Data	Original data	Non-redundant data	Balanced data
**S**	positive	2516	2506	2506
	
	negative	97965	87877	2506

**T**	positive	382	378	378
	
	negative	51434	10402	378

**Y**	positive	108	108	108
	
	negative	26405	1681	108

Redundant sequence fragments or sequences which contain the same amino acids on each corresponding position in both the positive data sets and negative data sets are removed. In the case of redundant sequence fragments found in both positive and negative data sets, the sequence in the negative data set is removed and the sequence in the positive data set is retained as shown in the redundancy removal step of Figure 1. The removal of redundant data resulted in 2506 positive fragments and 87877 negative fragments for S; 378 positive fragments and 10402 negative fragments for T; and 108 positive fragments and 1681 negative fragments for Y. Since the number of the negative fragments is much greater than the number of the corresponding positive fragments, the number of data is balanced by obtaining the same number of negative fragments based on the number of positive fragments. The idea of balancing the negative data with the positive data is done in reference to PhosPhAt [15] which uses random selection to obtain a balanced data set. However, in order to avoid an unstable prediction performance through a non-representative data set derived using random selection, the *K*-means clustering method [29] is used for this study. *K*-means clustering selects well-represented data from a large data set in order to achieve a more globalized sample. A data point which has a minimal distance from other data points surrounding it is selected as a representative data. For this study, *K*-means clustering is performed based on sequence identity. The value of *K *which denotes the number of samples to be obtained from the negative data set is defined by the number of the corresponding positive data. This resulted in a data set consisting of 2506 positive and negative S fragments; 378 positive and negative T fragments; and 108 positive and negative Y fragments. Table 1 shows the final number of positive and negative fragments in S, T, and Y used for this study.

### Data clustering by maximal dependence decomposition

One of the aims of this study is to investigate the substrate site specificity of plant phosphorylation sites based on amino acid sequences. In order to explore the conserved motifs from a large data set, MDD is applied to cluster all phosphorylated fragments into subgroups, which can show statistically significant site specificity. MDD [28] is a methodology to group a set of aligned signal sequences to moderate a large group into subgroups that capture the most significant dependencies between positions. In previous studies [28], MDD was proposed to group splice sites during the identification process of splice site prediction. However, in this work, we group protein sequences instead of nucleotides. MDD adopts chi-square test *χ*^2^(A_i_, A_j_) to evaluate the dependence of amino acid occurrence between two positions, *A_i _*and *A_j_*, which surround the phosphorylation site. In order to extract motifs that have conserved biochemical property of amino acids when doing MDD, we categorize the twenty types of amino acids into five groups: neutral, acid, basic, aromatic, and imino groups, as shown in Additional file 1, Table S1. Then, a contingency table of the amino acids occurrence between two positions is constructed, as presented in Additional file 1, Figure S1. The chi-square test is defined as:(1)

where *X_mn _*represents the number of sequences that have the amino acids of group *m *in position *A_i _*and have the amino acids of group *n *in position *A_j_*, for each pair (*A_i_, A_j_*) with *i*≠*j*. *E_mn _*is calculated as, where *X_mR _*= *X_m1_*+ ...+*X_m5_*, *X_Cn _*= *X_1n_*+ ...+*X_5n_*, and *X *denotes the total number of sequences. If a strong dependence is detected (defined as a *X^2 ^*value is larger than 34.3, corresponding to a cutoff level of *P *= 0.005 with 16 degrees of freedom) between two positions, then the process is continued as described by Burge and Karlin [28]. As illustrated in Additional file 1, Figure S1, it can be observed that position +1 has the maximal dependence with the occurrence of imino amino acids. Subsequently, all data can be divided into two subgroups where one has the occurrence of imino amino acids in position +1 while the other does not have the occurrence of imino amino acids in position +1. MDD clustering is a recursive process which divides the positive sets into tree-like subgroups. When applying MDD to cluster the sequences in the positive set, a parameter, i.e., the minimum-cluster-size, should be set. If the size of a subgroup is less than the minimum-cluster-size, the subgroup will not be divided any further. The MDD process terminates until all the subgroup sizes are less than the value of the minimum-cluster-size. With reference to previous works that utilize MDD [18], there exists no set values for the parameters of MDD clustering. In order to obtain an optimal minimum cluster size, MDD clustering is executed using various values. Each subgroup is represented using WebLogo [30] to graphically visualize the corresponding substrate motif. The resulting clusters are then analyzed as to whether or not they contain significant conserved motifs. Subgroups with very similar motifs are further grouped together into a single cluster in order to provide more meaningful groups and avoid redundant clusters as shown in the MDD clustering step of Figure 1.

### Model learning and evaluation

In this work, profile HMM is learned from the site sequences of each optimized MDD-clustered subgroups. An HMM describes a probability distribution over a potentially infinite number of sequences [31]. It can also be used to detect distant relationships between amino acids sequences. Here, we use the software package HMMER version 2.3.2 [31] to build the profile HMMs, to calibrate the HMMs, and to search the putative phosphorylation sites against the protein sequences. HMM builds a model based on positive instances of a class; thus, in this study, only positive data were utilized to build a model. The MDD-clustered sets of the phosphorylation sites are taken as training sets to learn the HMMs. One HMM is built for each MDD-clustered subgroup.

For each model of the MDD-clustered subgroups, a threshold parameter is selected as a cut-off value in identifying potential positive data from a query [31]. An optimized threshold is selected as the value which gives the most optimal cross-validation performance for each training model. To search the hits of a HMM, HMMER returns both a bit score and an expectation value (E-value). The bit score is the base two logarithm of the ratio between the probability that the query sequence is a significant match and the probability that it is generated by a random model. The E-value represents the expected number of sequences with a score greater than or equal to the returned HMMER bit scores. A search result with an HMMER bit score greater than the threshold parameter is taken as a positive prediction. While decreasing the bit score threshold favors finding true positives, increasing the bit score threshold favors finding true negatives.

Cross-validation is an important evaluation procedure prior to the application of a predictor [32]. The predictive performance of the constructed models is evaluated by performing five-fold cross validation. The training data is divided into five groups by splitting each dataset into five approximately equal sized subgroups. During cross-validation, each one of the five subgroups is regarded as the validation set in turn, and the remainder is regarded as the training set. Next, the following measures of predictive performance of the trained models are calculated: Precision (Pre) = TP/(TP+FP), Sensitivity (Sn) = TP/(TP+FN), Specificity (Sp) = TN/(TN+FP), and Accuracy (Acc) = (TP + TN)/(TP+FP+TN+FN), where TP, TN, FP and FN represent the numbers of true positives, true negatives, false positives and false negatives, respectively. Along with 5-fold cross-validation, different values for the HMMER bit score were also tested in order to obtain the optimal threshold parameter for predicting query sequences. Each value from -20 to 0 was each tested as the HMMER bit score. Then, the results of each fold using each bit score value is compared and analyzed. The value which yields the highest cross-validation performance on all five folds of a specific model is selected as the optimal HMMER threshold for that HMM.

### Independent testing

As for classification, the prediction performance of the trained models may be overestimated due to a possible over-fit in the training set; thus, an independent test is done. The experimental plant phosphorylation sites of UniProtKB/Swiss-Prot [11], which were not included in TAIR9 [17], are regarded as the independent test set and are used to estimate the actual prediction performance. Using a window size of 21-mer, there are 332, 105, and 14 phosphorylation sites for S, T, and Y, respectively. Similar to the extraction of a negative set of training data, there are 664, 210, and 28 non-phosphorylation sites for S, T, and Y, respectively, are regarded as the negative set of independent test data. A balanced number of negative data is selected using K-means clustering to match the number of positive data. After performing a five-fold cross-validation evaluation, the independent test set is used to evaluate the MDD-clustered HMMs with the highest accuracy. After searching against all HMMs, the prediction result for each test data on each HMM is evaluated. If a query data is predicted by at least one HMM to be positive, then it is reported by PlantPhos as a phosphorylation site. This is because each HMM represents the target motif of a specific plant protein kinase which means that a positive hit of a certain model matches the motif it represents. In case where a query data is predicted by two or more HMMs as a phosphorylation site, the cluster which gives the highest prediction score is treated as the plant protein kinase motif that the query data matches. Meanwhile, if a query data is predicted by all HMMs to be negative, then it is considered as a non-phosphorylation site. In order to justify the results due to the balancing of the positive and negative data, the independent testing is done 10 times on S, T, and Y data.

## Results and discussion

### Investigation of substrate site specificities

This work aims to investigate the various substrate site specificities in the plant species *Arabidopsis thaliana *based on amino acid sequences. According to a previous mass spectrometry-based identification of plant phosphorylation sites, a total of 2506, 378, and 108 non-redundant data for S, T, and Y, respectively, are extracted from the TAIR9 database [17] through its authors. As presented in Table 2, the Two Sample Logo [33] indicates that phosphoserine (pSer), phosphothreonine (pThr), and phosphotyrosine (pTyr) sites contain various conserved amino acids as opposed to non-phosphorylated S, T, and Y. In order to further investigate different substrate site specificities from the available data, this work applies MDD to cluster all phosphorylated fragments into subgroups that capture the most significant dependencies of amino acid composition between positions. Phosphorylated sequences in each MDD-clustered subgroup which shows a conserved motif, represents particular substrate site specificity. The flanking amino acids (-10 ~ +10) of the non-redundant phosphorylation sites, which are centered on position 0, are graphically visualized as sequence logos using WebLogo [30,34].

**Table 2:** Two Sample Logo in plant phosphoserine, phosphothreonine, and phosphotyrosine.

Phosphorylated Residue	Number of Non-redundant data	Entropy Plot of Sequence Logo
**S**	2506	

**T**	378	

**Y**	108	

Maximal dependence decomposition is executed multiple times with varying values in order to obtain the most optimal minimum cluster size. Setting the minimum cluster size to 200 for S data yielded 22 small groups. However, by further analyzing these groups through its corresponding entropy plots, it is observed that several groups contain very similar motifs. These groups are then combined together and visualized using WebLogo. The resulting entropy plots of the combined group exhibit the same motif as the smaller groups. Therefore, the groups were further tuned by combining similar groups into one group. This resulted in 9 subgroups for S. For T and Y data, the minimum cluster size was set to 100, and 30, respectively, which resulted to 6 subgroups containing distinct motifs for both data sets. The conservation of flanking amino acids in each MDD-clustered subgroup is represented using entropy plot of sequence logo. Table 3 shows the number of positive data in each subgroup of pSer and their corresponding predictive performances based on a five-fold cross-validation evaluation. According to the chi-square test of the dependence of five amino acid groups in flanking positions (see Additional file 1, Table S1), eight subgroups contain conserved motifs at a specific position. Subgroup S1 reveals that the most pronounced feature of plant pSer sites is the abundance of proline residue at position +1. Moreover, another significant substrate specificity is observed in subgroup S2 (786 pSer sequences) which contains a statistically conserved motif of negatively charged amino acids (D and E) at positions +3. Five out of all MDD-clustered subgroups contain conserved motifs of positively charged amino acids (K and R) at a specific position. In particular, subgroup S5 contains a conserved proline motif at position -9, which is distant to the phosphorylation site (position 0). Also, subgroups S3 and S8 contain conserved motifs at distant positions +9 and +10, respectively. It is observed that subgroup S9 does not contain significantly conserved amino acid at any position.

**Table 3:** The substrate site specificity and predictive performance in nine MDD-clustered subgroups of phosphoserine.

Group	Number of data	Entropy plot of substrate motif	HMMER bit score	Pre	Sn	Sp	Acc
**S1**	624		-3	89.8%	90.6%	89.7%	90.2%

**S2**	786		-4	76.6%	71.9%	78.1%	75.0%

**S3**	355		-7	72.1%	68.9%	73.2%	71.0%

**S4**	230		-6	90.3%	93.0%	90.0%	91.5%

**S5**	77		-12	76.9%	75.0%	74.3%	74.5%

**S6**	93		-10	90.2%	88.0%	90.4%	89.1%

**S7**	109		-8	90.8%	83.3%	91.7%	87.5%

**S8**	61		-12	77.7%	85.0%	80.2%	82.7%

**S9**	171		-6	79.6%	83.1%	78.5%	80.8%

**Average**				**82.6%**	**82.0%**	**82.9%**	**82.4%**

The MDD-clustered subgroups of pThr and pTyr are presented in Table 4 and 5, respectively. In plant pThr sites, subgroup T1 also has the abundance of proline residue at position +1. Another featured motif of pThr is in subgroup T3 which contains the statistically significant conservation of amino acid residues (D, E, N and Q) at positions +5. Additionally, subgroups T2, T4, and T5 contain conserved motifs at distant positions -7, +10, and +9, respectively. For plant pTyr sites, subgroup Y3 contains conserved motif of basic amino acids (K, R and H) at position -6; meanwhile, subgroups Y4 and Y5 contain conserved motifs of basic residues (K and R) and imino residue (P) at positions -1 and +2, respectively. Both T6 and Y6 do not contain significantly conserved amino acid at any position as observed in the entropy plot motif of Table 4 and Table 5, respectively. Although the application of MDD on 108 pTyr sequences could result in six subgroups, the number of data is deemed insufficient to investigate representative significant motifs. In order to further investigate S9 and T6, MDD clustering is re-applied on these subgroups. As shown in Additional file 1, Table S6, potential substrate specificities can be found within S9 and T6. However, the number of data on each potential substrate motif found is too small to support its validity. This may be improved as additional plant phosphorylation data are acquired.

**Table 4:** The substrate site specificity and predictive performance in six MDD-clustered subgroups of phosphothreonine.

Group	Number of data	Entropy plot of substrate motif	HMMER bit score	Pre	Sn	Sp	Acc
**T1**	43		-16	79.4%	74.1%	81.1%	77.7%

**T2**	88		-9	89.4%	91.9%	88.6%	90.3%

**T3**	77		-9	82.1%	80.3%	81.9%	81.1%

**T4**	34		-15	78.6%	76.6%	77.1%	76.5%

**T5**	42		-13	79.4%	71.1%	81.1%	75.9%

**T6**	94		-8	74.9%	61.0%	79.8%	70.5%

**Average**				**80.6%**	**75.8%**	**81.6%**	**78.6%**

**Table 5:** The substrate site specificity and predictive performance in six MDD-clustered subgroups of phosphotyrosine.

Group	Number of data	Entropy plot of substrate motif	HMMER bit score	Pre	Sn	Sp	Acc
**Y1**	11		-16	93.3%	90.0%	90.0%	90.0%

**Y2**	7		-14	80.0%	80.0%	100%	90.0%

**Y3**	15		-18	85.0%	90.0%	86.6%	85.0%

**Y4**	20		-17	90.0%	90.0%	90.0%	90.0%

**Y5**	16		-16	90.0%	86.6%	88.3%	87.1%

**Y6**	39		-16	93.3%	92.1%	92.5%	92.4%

**Average**				**88.6%**	**88.1%**	**91.2%**	**89.0%**

### Predictive performance of five-fold cross-validation

The cross-validation process includes the selection of the threshold parameter for each model. The threshold parameter is a specific bit score that serves as the cutoff value of HMMsearch for determining matching query sequences for an HMM [31]. The threshold is selected by first testing each value from the range of -20 to 0 as the bit score. The threshold is tuned to a specific value which allows an HMM to yield a high and balanced Specificity and Sensitivity for a specific HMM. Table 3 shows the threshold score selected for each model of pSer as well as its individual predictive performance. Also, Table 4 and 5 show the threshold score selected for each model of pThr and pTyr, respectively, as well as its individual predictive performance.

According to a five-fold cross-validation evaluation, the predictive performance of MDD-clustered HMM performs significantly better than the non-MDD-clustered HMM of pSer, pThr, and pTyr. As shown in Table 6, the single HMM for pSer yields a Precision rate of 49.5%, a Sensitivity rate of 58.6%, a Specificity rate of 70.0%, and an Accuracy rate of 66.2%. On the other hand, the performance of HMM for pSer with MDD clustering yields a Precision rate of 82.6%, a Sensitivity rate of 82.0%, a Specificity rate of 82.9%, and an Accuracy rate of 82.4%. With regard to pThr, using a single HMM yields a Precision rate of 45.4%, a Sensitivity rate of 60.5%, a Specificity rate of 63.4%, and an Accuracy rate of 62.5%. On the other hand, the performance of MDD-clustered HMM for pThr yields a Precision rate of 80.6%, a Sensitivity rate of 75.8%, a Specificity rate of 81.6%, and an Accuracy rate of 78.6%. With regard to pTyr, using a single HMM yields a Precision rate of 75.4%, a Sensitivity rate of 90.6%, a Specificity rate of 84.7%, and an Accuracy rate of 86.6%. On the other hand, the performance of HMM for pTyr with MDD clustering yields a Precision rate of 88.6%, a Sensitivity rate of 88.1%, a Specificity rate of 91.2%, and an Accuracy rate of 89.0%. This results show that HMM for pSer, pThr, and pTyr which utilizes MDD performs significantly better than using a single HMM without MDD clustering.

**Table 6:** The comparison of five-fold cross-validation between single HMM and MDD-clustered HMMs.

Method	Phosphorylated residue	Pre	Sn	Sp	Acc
**Single HMM**	S	49.5%	58.6%	70.0%	66.2%
	
	T	45.4%	60.5%	63.4%	62.5%

	Y	75.4%	90.6%	84.7%	86.6%
	
**MDD-clustered HMMs**	S	82.6%	82.0%	82.9%	82.4%
	
	T	80.6%	75.8%	81.6%	78.6%
	
	Y	88.6%	88.1%	91.2%	89.0%

### Evaluation of the selected models using independent test set

Owing to a possible over-fit of the training set used in PlantPhos, the method was further evaluated by using an independent data set. The independent testing data was tested on each HMM model using the selected threshold score. Independent testing is done 10 times on pSer, pThr, and pTyr data in consideration for the balancing of the positive and negative testing data. The independent performance of each pSer model is shown on Additional file 1, Table S2; each pThr model on Additional file 1, Table S3; and each pTyr model on Additional file 1, Table S4. Moreover, as shown in Table 7, the method yields a significantly high sensitivity rate with 81.4% for MDD-clustered pSer models; 77.1% for MDD-clustered pThr models; and 83.7% for MDD-clustered pTyr models. On the other hand, it is also observed that the method yields slightly low specificity on the independent test data with a rate of 71.6% for MDD-clustered pSer models, 69.7% for MDD-clustered pThr models, and 68.7% for MDD-clustered pTyr models. The slightly high rate of False Positive predictions could be explained by the number of models we have for pSer, pThr, and pTyr. It is observed that although each model yields a high predictive specificity (90%), the existence of a large number of models may induce higher false positive predictions (see Additional file 1, Figure S2). Without the annotation of the corresponding kinase on each phosphorylation site, each query data is predicted based on observed substrate site specificities represented by MDD-clustered HMMs. As discussed above, if a query data is predicted by at least one of MDD-clustered HMMs to be positive, then it is reported by PlantPhos as a potential phosphorylation site. Meanwhile, if a query data is predicted by all HMM models to be negative, then it is considered as a non-phosphorylation site. Thus, a high false positive rate may be yielded by the prediction system. Furthermore, reasonably assuming that each MDD-clustered HMM used in PlantPhos represents the target motif of a plant protein kinase, a false positive prediction may be considered as a novel phosphorylation site. This is due to the fact that the negative data used in this study are not necessarily non-phosphorylation sites although it has not yet been experimentally identified to be phosphorylation sites.

**Table 7:** The comprison of independent testing.

Method	Phosphorylated residue	Pre (%)	Sn (%)	Sp (%)	Acc (%)
**Single HMM**	S	52.6%	50.6%	77.2%	68.3%
	
	T	50.0%	20.9%	89.5%	66.6%
	
	Y	36.3%	28.5%	75.0%	59.5%

**MDD-clustered HMMs**	S	74.2%	81.4%	71.6%	76.5%
	
	T	71.8%	77.1%	69.7%	73.4%
	
	Y	72.8%	83.7%	68.7%	76.2%

**PhosPhAt 3.0**	S	55.5%	61.9%	50.6%	56.2%
	
	T	55.0%	57.1%	53.3%	55.2%
	
	Y	4.0%	14.2%	78.5%	46.4%

**NetPhos 2.0**	S	58.6%	71.6%	49.3%	60.5%
	
	T	47.5%	36.1%	59.6%	47.8%
	
	Y	44.4%	28.5%	64.2%	46.4%

**DisPhos 1.3**	S	66.8%	60.6%	69.3%	65.0%
	
	T	47.5%	36.1%	61.2%	48.6%
	
	Y	57.1%	47.5%	64.2%	55.8%

### Comparison with other methods

Currently, very few prediction methods dedicated to identifying phosphorylation sites in plants have been proposed. Among these are PhosPhAt [15] which uses a SVM classifier and the work of Gao *et al. *[26] which incorporates protein sequence information, protein disordered regions, integrated k-nearest neighbor, and SVM for predicting phosphorylation sites (see Additional file 1, Table S5). However, only PhosPhAt offers a readily-available web tool, PhosPhAt 3.0, for predicting plant phosphorylation sites. For comparison, the independent test data set used for evaluating PlantPhos was utilized. Using a window size of 21-mer, 332 pSer, 105pThr, and 14 pTyr sites were used as the positive test data. A balanced number of negative data is then selected using K-means clustering to match the number of positive data. The resulting data are then classified as phosphorylation or non-phosphorylation sites using both PlantPhos and PhosPhAt 3.0. Finally, the precision, sensitivity, specificity, and accuracy of both methods are calculated in order to compare their respective predictive performance. Table 7 shows the results of both methods after being tested on the same data set. It can be observed that PhosPhAt 3.0 yields an accuracy of 56.2%, 55.2%, and 46.4% for predicting S, T, and Y sites, respectively, which is lower as compared to the proposed method which yields an accuracy of 76.5%, 73.4%, 76.2% for predicting S, T, and Y sites, respectively.

Moreover, the predictive performance of PlantPhos is compared with general phosphorylation prediction tools in order to compare its ability in identifying plant phosphorylation sites. NetPhos is a general phosphorylation prediction tool trained on mammalian phosphoproteins which uses an artificial neural network method to predict phosphorylation sites in query sequences [35]. DisPhos is another general phosphorylation prediction tool trained on experimentally verified phosphorylation sites which uses disorder information to improve prediction accuracy [36]. Furthermore, DisPhos provides an *Arabidopsis thaliana *predictor, which is selected for this test. These two phosporylation tools, which are available online, are tested using the same independent data set used for evaluating PlantPhos. Table 7 shows that NetPhos yields an accuracy of 60.5%, 47.8%, and 46.4% for predicting S, T, and Y sites. On the other hand, DisPhos 1.3 [36] yields an accuracy of 65.0%, 48.6%, and 55.8% for predicting S, T, and Y sites.

### Comparison of motifs between MDD-clustered subgroups and well-known kinase groups

In order to further investigate the significance of each MDD-generated motif that represents its corresponding substrate specificity, each MDD-generated motif is compared with the motifs of known kinase-specific motifs. In order to obtain known kinase-specific motifs, all available kinase-specific phosphorylation sites of multiple organisms were obtained from Phospho.ELM [9] and were integrated together to generate a set of known kinase-specific motifs. The resulting MDD motifs generated in this study were then mapped to the kinase-specific motifs generated from the available data in Phospho.ELM. According to the chi-square test of the dependence of five amino acid groups in flanking positions of plant pSer, the most featured motif is the group that contains conserved proline (P) at position +1, which is highly similar to two well-known kinase groups in Phospho.ELM, Mitogen Activated Protein Kinase (MAPK) and Cyclin Dependent Kinase (CDK). From the pSer subgroups, it is observed that S1 shows a conserved P at position +1; thus, it can be matched with MAPK and CDK. Furthermore, it is observed that S2 contains a conserved Glutamic Acid (G) and Aspartic Acid (D) at positions +1, +2 and +3. A similar conservation is seen in the Casein kinase II (CK2) family of non-plant organisms in Phospho.ELM. Thus, the pSer group S2 can be matched with CK2. Additionally, the conserved Arginine (A) and Lysine (K) at position -3 in the pSer group S4 is observed to be similar with the CAMK family 2 (CAMK2) of non-plant organisms in Phospho.ELM; therefore, S4 can be matched with CAMK2. Overall, as presented in Table 8, plant pSer groups S1 is matched to kinase groups MAPK and CDK, as well as S2 and S4 are matched to kinase groups CK2 and CAMK2, respectively. Similar to the matches in pSer subgroup S1, pThr subgroups T1 is matched to kinase groups MAPK and CDK for having similar conserved amino acids at the same position. Additionally, subgroup T3 is matched to the kinase group CK2.

**Table 8:** The MDD-clustered subgroups matched to the well-known kinase groups in non-plant organisms of Phospho.ELM.

Group	Number of data	Entropy plot of motif	Matched kinase	Entropy plot of kinase motif
**S1**	624		MAPK	

			CDK	

**S2**	786		CK2	

**S4**	230		CAMK2	

**T1**	43		MAPK	

			CDK	

**T3**	77		CK2	

### Implementation of the prediction scheme

**Figure 2:**
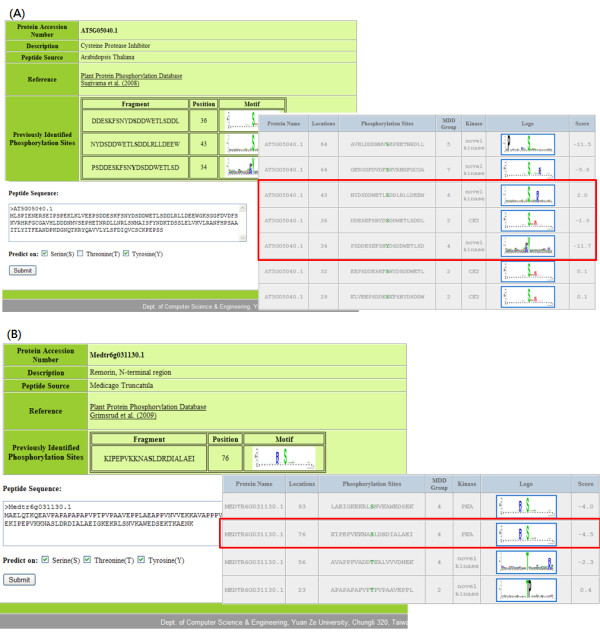
**Cases study of (A) protein AT5G05040.1 from *Arabidopsis Thaliana *and (B) protein Medtr6g031130.1 from *Medicago Truncatula***.

Due to the time-consuming and laboratory-intensive nature of experimental identification, even though a protein can be phosphorylated, precisely identifying the phosphorylation sites on the plant substrate proteins is difficult. Therefore, an effective prediction tool should be developed to identify phosphorylation sites with its substrate site specificity. Following the evaluation by cross-validation and independent testing, the MDD-clustered HMMs are utilized in the construction of web-based prediction system. Users can submit their uncharacterized protein sequences in the system; consequently, the web tool, PlantPhos, returns the predicted sites together with the position of the predicted site, the flanking amino acids, the matched MDD-clustered substrate motif, and the prediction score. To demonstrate the performance of the proposed tool, two experimentally verified phosphorylated plant proteins which do not exist in the training data set was studied. The first case study is performed using AT5G05040.1 which is a Cysteine protease inhibitor protein from *Arabidopsis thaliana *obtained from the Plant Protein Phosphorylation Database [16]. As shown in Figure 2(A), AT5G05040.1 contains 2 experimentally verified pSer at positions 36 and 43, and an experimentally verified pTyr at position 34. PlantPhos is able to correctly predict the experimentally verified pSer at positions 36 and 43 as well as the experimentally verified pTyr at position 34. Moreover, three more S residues are reported by PlantPhos as novel phosphorylated sites. Next, in order to evaluate the performance of PlantPhos in identifying phosphorylation sites from a different plant species aside from *Arabidopsis thaliana*, the second case study is performed using Medtr6g031130.1 which is a Remorin, N-terminal region protein from *Medicago Truncatula *obtained from the Plant Protein Phosphorylation Database [16]. As shown in Figure 2(B), Medtr6g031130.1 contains 1 experimentally verified pSer at positions 76. PlantPhos is able to correctly predict the experimentally verified pSer at position 76. Additionally, one more S and two T are reported by PlantPhos as novel phosphorylated sites.

## Conclusions

The importance of phosphorylation has been indicated in the regulation of protein functions and cell signaling, but the state of research in this field is hindered by experimental difficulties especially for the investigation of substrate site specificity in various organisms. In this work, we have analyzed plant phosphorylation sites by applying MDD clustering; using this method, the available plant phosphorylation data were grouped into several subgroups-each showing a significant conserved motif. Then, we developed a novel method for predicting protein phosphylation sites in plants by training HMMs for each MDD-clustered subgroup which are then used to predict potential phosphorylation sites by HMMsearch. Our method is evaluated by means of 5-fold cross-validation which yields an average accuracy of 82.4% for predicting pSer, 78.6% for predicting pThr, and 89.0% for predicting pTyr. Moreover, our method is further evaluated by testing it on an independent data set which shows that our method can predict novel phosphorylation sites by using the experimental phosphorylation data in plant proteins from UniProtKB/SwissProt. Additionally, we were able to further investigate the MDD-clustered motifs in plants by referencing to the motifs of known kinases from Phospho.ELM [9]. Through this method, we were able to observe similar kinase motifs between plants and other organisms. Lastly, the method has been implemented as a web tool named PlantPhos in order for the research community to be able to facilitate phosphorylation site prediction on plant protein data using the proposed method.

Future development for PlantPhos involves (i) the extension of the system to include other plant species other than *Arabidopsis thaliana*; (ii) the acquisition of additional experimentally verified plant protein data to re-calibrate more robust MDD clusters; (iii) and a more comprehensive investigation of substrate site specificities in plants with additional plant phosphorylation data.

## Availability

PlantPhos can be accessed via a web interface, and is freely available to all interested users at http://csb.cse.yzu.edu.tw/PlantPhos/. All of the data set used in this work is also available for download in the website.

## Competing interests

The authors declare that they have no competing interests.

## Authors' contributions

TYL conceived and supervised the project. NAB was responsible for the design, computational analyses, implemented the web-based tool, and drafted the manuscript with revisions provided by TYL and CTL. All authors read and approved the final manuscript.

## Supplementary Material

Additional file 1 **Additional Figures and Tables**. Contains additional Figures and Tables showing further results in the study Click here for file
