# Supplementary material for: PlantPhos: using maximal dependence decomposition to identify plant phosphorylation sites with substrate site specificity
Source: BMC Bioinformatics. 2011 Jun 26;12:261. doi: 10.1186/1471-2105-12-261 (PMC3228547; doi:10.1186/1471-2105-12-261)
Supplement: Additional file 1 — Additional Figures and Tables. Contains additional Figures and Tables showing further results in the study [file 1471-2105-12-261-S1.DOC]

**Additional File 1**

**Figures**


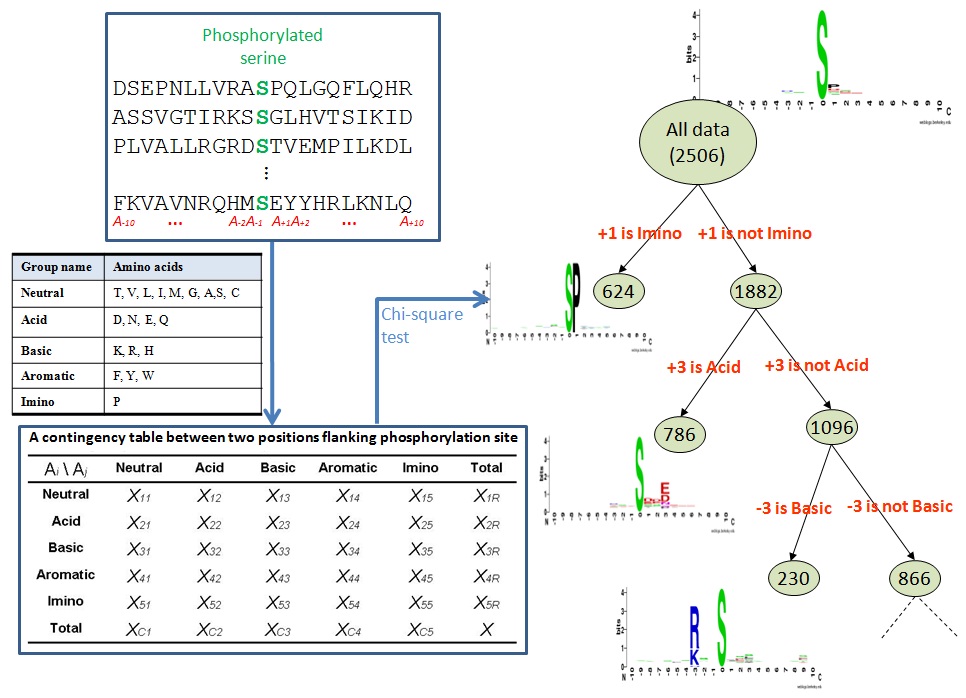


**Figure S1**. Analytical flowchart of MDD.

**
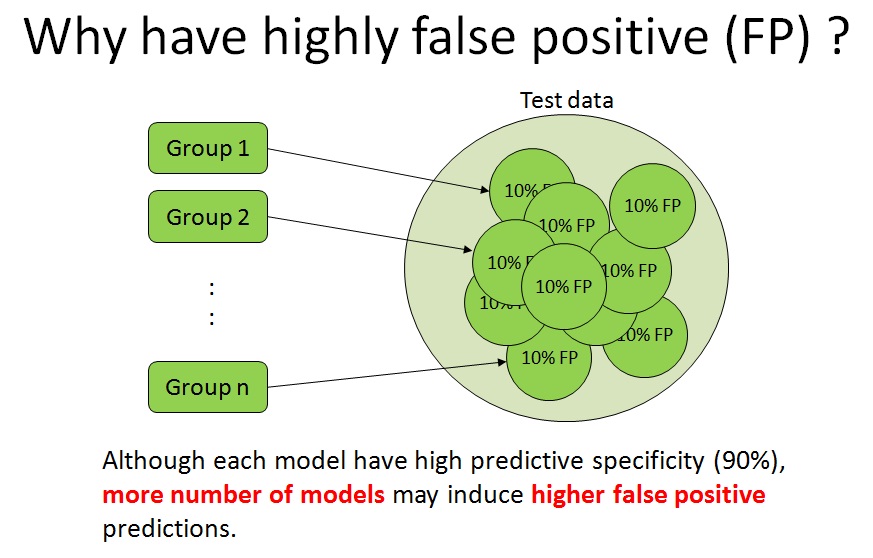
**

**Figure S2**. A conceptual figure explaining why there is a highly false positive prediction rate.

**Tables**

**Table S1.** Amino acid groups used in MDD clustering

| **Group name** | **Amino acids** |
| --- | --- |
| **Neutral** | Threonine (T), valine (V), leucine (L), isoleucine (I), methionine (M), glycine (G), alanine (A), serine (S), cysteine (C) |
| **Acid** | Aspartic acid (D), asparagine (N), glutamic acid (E), glutamine (Q) |
| **Basic** | Lysine (K), arginine (R), histidine (H) |
| **Aromatic** | Phenylalanine (F), tyrosine (Y), tryptophan (W) |
| **Imino** | Proline (P) |

**Table S2.** Predictive performance for 10 random tests on an independent phosphoserine testing data

| **Test data** | **Pre** | **Sn** | **Sp** | **Acc** |
| --- | --- | --- | --- | --- |
| **1** | 76.20% | 82.30% | 74.30% | 78.30% |
| **2** | 77.97% | 80.29% | 77.32% | 78.81% |
| **3** | 75.07% | 83.33% | 72.33% | 77.83% |
| **4** | 73.47% | 84.00% | 69.66% | 76.83% |
| **5** | 73.38% | 76.62% | 72.20% | 74.41% |
| **6** | 75.00% | 82.10% | 72.64% | 77.37% |
| **7** | 71.19% | 80.72% | 67.33% | 74.03% |
| **8** | 76.35% | 82.65% | 74.40% | 78.53% |
| **9** | 72.38% | 79.68% | 69.60% | 74.64% |
| **10** | 71.20% | 82.11% | 66.78% | 74.45% |
| **Average** | **74.22%** | **81.38%** | **71.66%** | **76.52%** |

**Table S3.** Predictive performance for 10 random tests on an independent phosphothreonine testing data.

| **Test data** | **Pre** | **Sn** | **Sp** | **Acc** |
| --- | --- | --- | --- | --- |
| **1** | 71.42% | 78.21% | 68.70% | 73.46% |
| **2** | 71.82% | 77.64% | 69.54% | 73.59% |
| **3** | 72.65% | 73.51% | 72.33% | 72.92% |
| **4** | 72.56% | 80.22% | 69.66% | 74.94% |
| **5** | 73.02% | 80.33% | 70.32% | 75.33% |
| **6** | 71.32% | 75.64% | 69.58% | 72.61% |
| **7** | 69.10% | 74.32% | 66.76% | 70.54% |
| **8** | 74.98% | 81.23% | 72.90% | 77.07% |
| **9** | 73.14% | 77.87% | 71.40% | 74.64% |
| **10** | 68.48% | 72.68% | 66.54% | 69.61% |
| **Average** | **71.85%** | **77.17%** | **69.77%** | **73.47%** |

**Table S4. Predictive performance for 10 random tests on an independent phosphotyrosine testing data.**

| **Test data** | **Pre** | **Sn** | **Sp** | **Acc** |
| --- | --- | --- | --- | --- |
| **1** | 71.42% | 86.32% | 65.45% | 75.89% |
| **2** | 73.03% | 84.21% | 68.90% | 76.56% |
| **3** | 74.94% | 83.33% | 72.13% | 77.73% |
| **4** | 74.08% | 84.44% | 70.45% | 77.45% |
| **5** | 73.57% | 84.44% | 69.66% | 77.05% |
| **6** | 75.00% | 82.10% | 72.64% | 77.37% |
| **7** | 71.54% | 82.13% | 67.33% | 74.73% |
| **8** | 73.50% | 86.54% | 68.80% | 77.67% |
| **9** | 70.46% | 82.41% | 65.45% | 73.93% |
| **10** | 71.07% | 81.62% | 66.78% | 74.20% |
| **Average** | **72.86%** | **83.75%** | **68.76%** | **76.26%** |

**Table S5**. Comparison between PlantPhos and other methods.

| **Tool** | | |  | **PlantPhos** | **PhosPhAt** | **Gao *et al.*** |
| --- | --- | --- | --- | --- | --- | --- |
| Materials | | |  | TAIR9 + SwissPRot | PhosPhAt | PhosPhAt + TAIR |
| Method | | |  | profile HMM + MDD | SVM | SVM |
| Training features | | |  | Amino acid sequences | Amino acid sequences | Amino acid sequences + disorder |
| Cross-validation | | Sensitivity | | 82.0% | ~81% | ~80% |
| on Serine | | Specificity | | 82.9% | ~60% | ~80% |
|  | | Accuracy | | 82.4% | - | - |
|  | | Precision | | 82.6% | - | - |
| Cross-validation | | Sensitivity | | 75.8% | ~69% | - |
| on Threonine | | Specificity | | 81.6% | ~50% | - |
|  | | Accuracy | | 78.6% | - | - |
|  | | Precision | | 80.6% | - | - |
| Cross-validation | | Sensitivity | | 88.1% | ~65% | - |
| On Tyrosine | | Specificity | | 91.2% | ~50% | - |
|  | | Accuracy | | 89.0% | - | - |
|  | Precision | | | 88.6% | - | - |

**Table S6**. Further MDD clustering results for subgroups S9 and T6

| **Group** | **Sub-group** | **Entropy Plot of Potential Substrate Motif** | **Number**  **of Data** |
| --- | --- | --- | --- |
| **S9** | S9.1 | 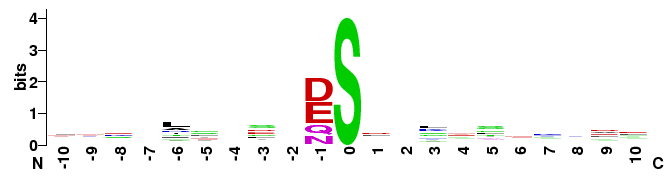 | 39 |
|  | S9.2 | 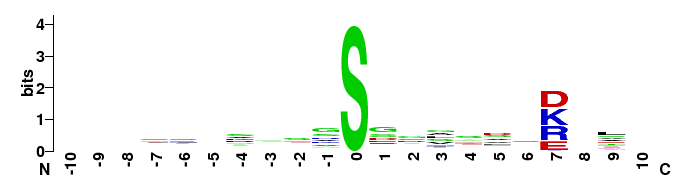 | 44 |
|  | S9.3 | 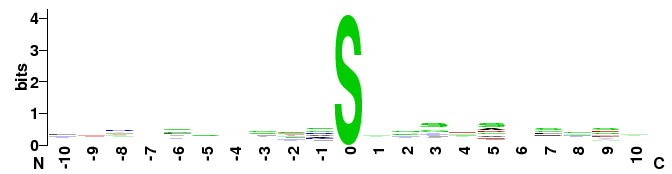 | 88 |
| **T6** | T6.1 | 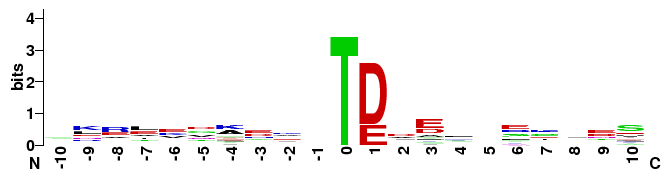 | 16 |
|  | T6.2 | 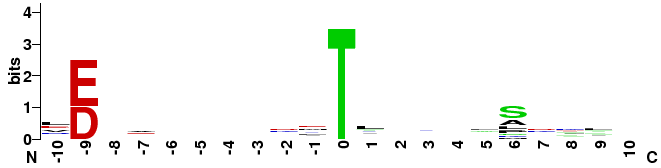 | 17 |
|  | T6.3 | 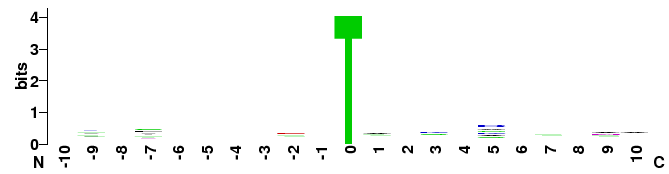 | 61 |
